# Supplementary material for: Validation of improved cytochrome c oxidase I (COI) primers for comprehensive biodiversity assessment of ascidians
Source: PeerJ. 2025 Jul 14;13:e19671. doi: 10.7717/peerj.19671 (PMC12269779; doi:10.7717/peerj.19671)
Supplement: Supplemental Information 1 — Polymerase chain reaction (PCR) amplification results are indicated by a circle ($○$○) for a successful reaction and a dash (–) for a failed reaction. [file peerj-13-19671-s001.docx]

| Lane no. | Phylum | Class | Order | Family | Scientific name | PCR amplification |
| --- | --- | --- | --- | --- | --- | --- |
| 1 | Chordata | Ascidiacea | Aplousobranchia | Didemnidae | *Didemnum vexillum* | ○ |
| 2 |  |  |  |  | *Ascidiella aspersa* | ○ |
| 3 |  |  |  | Cionidae | *Ciona robusta* | ○ |
| 4 |  |  |  |  | *Ciona savignyi* | ○ |
| 5 |  |  | Stolidobranchia | Styelidae | *Styela plicata* | ○ |
| 6 |  |  |  | Pyuridae | *Herdmania momus* | ○ |
| 7 | Echinodermata | Crinoidea | Comatulida | Colobometridae | *Decametra tigrina* | – |
| 8 |  | Asteroidea | Valvatida | Asterinidae | *Patiria pectinifera* | – |
| 9 |  | Echinoidea | Camarodonta | Strongylocentrotidae | *Mesocentrotus nudus* | – |
| 10 | Bryozoa | Gymnolaemata | Cheilostomatida | Watersiporidae | *Watersipora subtorquata* | – |
| 11 |  |  |  | Bugulidae | *Bugula neritina* | – |
| 12 |  |  |  |  | *Bugulina californica* | – |
| 13 |  |  |  | Candidae | *Caberea lata* | – |
| 14 | Arthropoda | Thecostraca | Balanomorpha | Balanidae | *Fistulobalanus albicostatus* | – |
| 15 |  |  |  |  | *Amphibalanus improvisus* | – |
| 16 |  |  |  |  | *Amphibalanus eburneus* | – |
| 17 |  |  |  |  | *Balanus trigonus* | – |
| 18 | Mollusca | Bivalvia | Ostreida | Ostreidae | *Magallana gigas* | – |
| 19 |  |  | Mytilida | Mytilidae | *Mytilus galloprovincialis* | – |
| 20 | Anthozoa | Hexacorallia | Actiniaria | Actiniidae | *Anthopleura fuscoviridis* | – |
| 21 | Porifera | Demospongiae | Suberitida | Halichondriidae | *Halichondria (Halichondria) bowerbanki* | – |
| 22 |  |  |  |  | *Hymeniacidon perlevis* | – |
