# Supplementary material for: Validation of improved cytochrome c oxidase I (COI) primers for comprehensive biodiversity assessment of ascidians
Source: PeerJ. 2025 Jul 14;13:e19671. doi: 10.7717/peerj.19671 (PMC12269779; doi:10.7717/peerj.19671)
Supplement: Supplemental Information 2 [file peerj-13-19671-s002.docx]

| Coefficients | Estimate | Std. Error | z value | *p* |
| --- | --- | --- | --- | --- |
| AscCOI (Intercept) | -0.0550 | 0.1510 | -0.3644 | 0.7155 |
| AscCOI2 | 2.0206 | 0.2611 | 7.7373 | 0.0000 |
