# Supplementary material for: Validation of improved cytochrome c oxidase I (COI) primers for comprehensive biodiversity assessment of ascidians
Source: PeerJ. 2025 Jul 14;13:e19671. doi: 10.7717/peerj.19671 (PMC12269779; doi:10.7717/peerj.19671)
Supplement: Supplemental Information 3 [file peerj-13-19671-s003.docx]

| Coefficients | Estimate | Std. Error | z value | *p* |
| --- | --- | --- | --- | --- |
| AscCOI (Intercept) | 10.7130 | 0.6906 | 15.5115 | 0.0001 |
| AscCOI2 | 14.9600 | 1.1362 | 13.1664 | 0.0001 |
