# Supplementary material for: Validation of improved cytochrome c oxidase I (COI) primers for comprehensive biodiversity assessment of ascidians
Source: PeerJ. 2025 Jul 14;13:e19671. doi: 10.7717/peerj.19671 (PMC12269779; doi:10.7717/peerj.19671)
Supplement: Supplemental Information 4 — Only the binding success rate of the primer pairs (percentage of templates with both forward and reverse primers successfully bound) is shown. [file peerj-13-19671-s004.docx]

| Taxon | Species | |  | Sequence | |
| --- | --- | --- | --- | --- | --- |
|  | Total | Binding (%) |  | Total | Binding (%) |
| Anthozoa | 1,469 | 58 (3.95) |  | 8,449 | 431 (5.10) |
| Bivalvia | 472 | 11 (2.33) |  | 2,917 | 17 (0.58) |
| Gymnolaemata | 217 | 24 (11.06) |  | 1,810 | 68 (3.76) |
| Hydrozoa | 547 | 0 (0.00) |  | 4,461 | 0 (0.00) |
| Porifera | 1,099 | 1 (0.09) |  | 6,594 | 1 (0.02) |
| Thecostraca | 361 | 2 (0.55) |  | 14,122 | 4 (0.03) |
| Echinodermata | 1,565 | 0 (0.00) |  | 19,905 | 0 (0.00) |
| Chondrichthyes | 924 | 0 (0.00) |  | 20,173 | 0 (0.00) |
