# Supplementary figures and images for: Validation of improved cytochrome c oxidase I (COI) primers for comprehensive biodiversity assessment of ascidians
Source: PeerJ. 2025 Jul 14;13:e19671. doi: 10.7717/peerj.19671 (PMC12269779; doi:10.7717/peerj.19671)

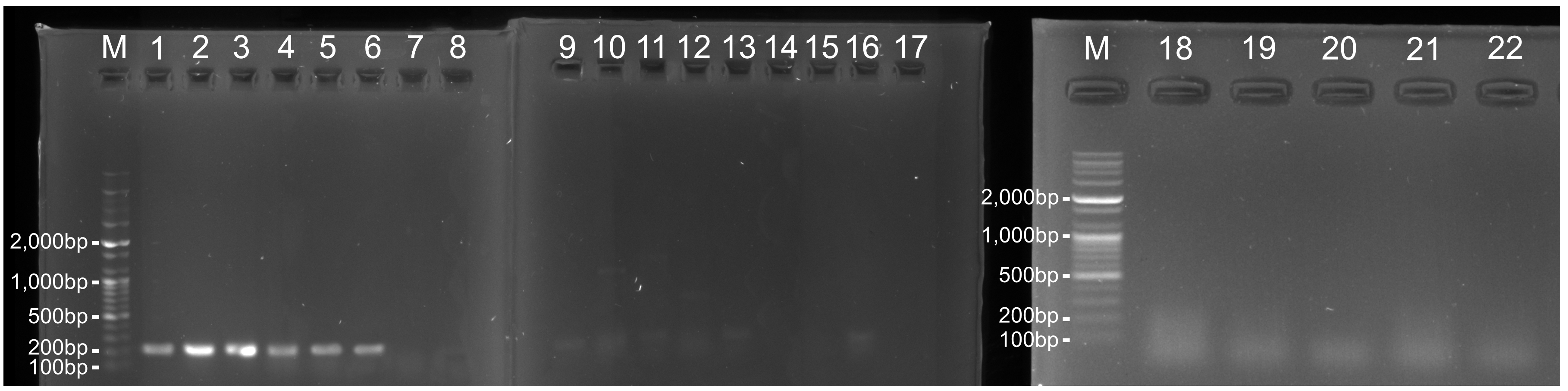

Supplement: Supplemental Information 5 — Lane M: molecular marker (100 –10,200 bp DNA ladder), Lanes 1–6: PCR products of ascidian (1: Didemnum vexillum, 2: Ascidiella aspersa, 3: Ciona robusta, 4: Ciona savignyi, 5: Styela plicata, 6: Herdmania momus), Lanes 7 –22: PCR products of marine benthic invertebrate (7: Decametra tigrina, 8: Patiria pectinifera, 9: Mesocentrotus nudus, 10: Watersipora subtorquata, 11: Bugula neritina, 12: Bugulina californica, 13: Caberea lata, 14: Fistulobalanus albicostatus, 15: Amphibalanus improvisus, 16: Amphibalanus eburneus, 17: Balanus trigonus, 18: Magallana gigas, 19: Mytilus galloprovincialis, 20: Anthopleura fuscoviridis, 21: Halichondria (Halichondria) bowerbanki, 22: Hymeniacidon perlevis). Only target group (Lanes 1–6) showed positive PCR bands. [file peerj-13-19671-s005.png]

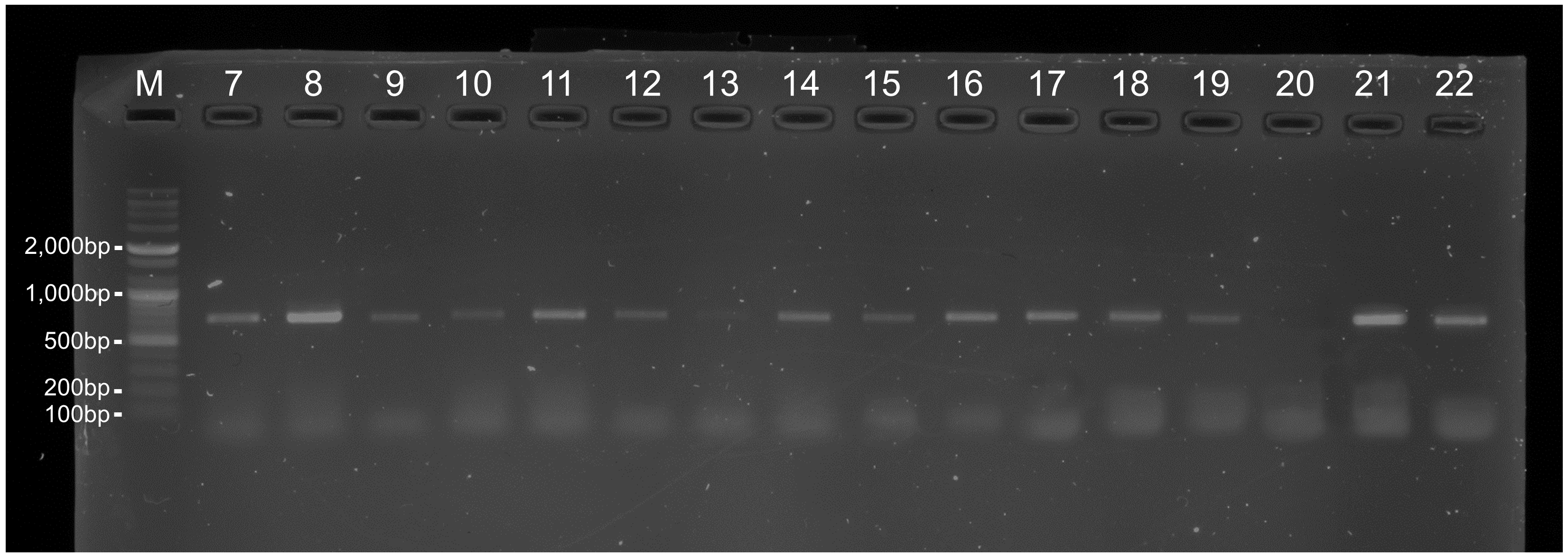

Supplement: Supplemental Information 6 — Lane M: molecular marker (100–10,200 bp DNA ladder). Lanes 7–22: PCR products of marine benthic invertebrates (same order as in Table S2 and Fig. S1). [file peerj-13-19671-s006.png]
